# Adhesion of Human Probiotic *Lactobacillus rhamnosus* to Cervical and Vaginal Cells and Interaction with Vaginosis-Associated Pathogens

**DOI:** 10.1155/2008/549640

**Published:** 2009-01-27

**Authors:** Sophie Coudeyras, Gwendoline Jugie, Marion Vermerie, Christiane Forestier

**Affiliations:** Laboratory of Bacteriology, UFR Pharmacy, University Clermont 1, 28 place H. Dunant, 63000 Clermont-Ferrand, France

## Abstract

*Objectives*. The ability of a probiotic *Lactobacillus rhamnosus* strain (Lcr35) to adhere to cervical and vaginal cells and to affect the viability of two main vaginosis-associated pathogens, *Prevotella bivia, Gardnerella vaginalis*, as well as *Candida albicans* was investigated. *Methods*. Adhesion ability was determined in vitro with immortalized epithelial cells from the endocervix, ectocervix, and vagina. Coculture experiments were performed to count viable pathogens cells in the presence of Lcr35. *Results*. Lcr35 was able to specifically and rapidly adhere to the three cell lines. In coculture assays, a decrease in pathogen cell division rate was observed as from 4 hours of incubation and bactericidal activity after a longer period of incubation, mostly with *P. bivia*. *Conclusion*. The ability of Lcr35 to adhere to cervicovaginal cells and its antagonist activities against vaginosis-associated pathogens suggest that this probiotic strain is a promising candidate for use in therapy.

## 1. INTRODUCTION

Bacterial vaginosis (BV) is the most frequent vaginal infectious disorder in women of childbearing age with prevalences ranging from 10% to 50% [1]. In addition to the physiological burden that induces BV, it can cause serious sequelae such as preterm birth and facilitate the acquisition of sexually transmitted diseases. The cause of BV remains poorly understood, and no specific infectious agents have been identified. However, the disorder is characterized by modifications of the genital tract microflora, including a reduction in or absence of lactobacillus colonization and overgrowth of several anaerobic bacteria [2]. The vaginal ecosystem in healthy premenopausal women harbors a microbiota dominated by *Lactobacilli* [3, 4], that is, being increasingly recognized as protecting it from invading pathogens, including those that cause urinary tract infections and sexually transmitted diseases. Different mechanisms are potentially involved in the activity of *Lactobacilli* against pathogens, including the competitive exclusion of genitourinary pathogens from receptors present on the surface of the epithelial cells. Under healthy conditions, cervicovaginal cells are constantly exposed to the normal vaginal microbiota.

The recommended treatment regimens for vaginal infections are oral or intravaginal antibiotics [5], but these conventional treatments are associated with frequent recurrences. Alternative therapeutic agents need to be sought, and it has been suggested that the administration of *Lactobacilli* can restore ecological balance in the vagina by controlling the infectivity of pathogenic microbes [3], but the treatment is still a subject of debate. Several clinical trials have been performed to investigate the effects of specific strains, mainly with *L. acidophilus* and *L. rhamnosus* species [6, 7], but no definitive conclusions as to whether these probiotics represent an effective and safe method for treating women with BV can be drawn. The behavior of the probiotics in the vaginal tract is likely to be strain specific and therefore, it is important to determine the characteristics of the strain to be used as a therapeutic agent. The most relevant properties in this context are likely to be adhesion to cervicovaginal cells and adequate pathogen growth inhibition. In vitro studies assessing these properties might not be able to fully simulate the in vivo behavior, but they could be reliable indicators when selecting the probiotic strain. The purpose of this study was to determine the in vitro adherence of a well characterized *L. rhamnosus* probiotic strain, Lcr35 [8–10], and its ability to inhibit growth of three vaginosis-associated pathogens. We used immortalized morphologically and functionally distinct epithelial cell lines from normal endocervix, ectocervix, and vagina to characterize Lcr35 epithelial interactions pertinent to the lower female genital tract and determined its antimicrobial activity against *Prevotella bivia*, *Gardnerella vaginalis*, and *Candida albicans* in coculture experiments.

## 2. MATERIALS AND METHODS

### 2.1. Adhesion assay

Adhesion assays were performed with epithelial cells from normal human vagina (VK2/E6E7 ATCC-CRL-2616), ectocervix (Ect1/E6E7 ATCC-CRL-2614), and endocervix (End1/E6E7 ATCC-CRL-2615), immortalized by expression of the E6 and E7 genes of human papillomavirus type 16 [11]. The morphological and immunocytochemical characteristics of the immortalized lines closely resembled those of their tissues of origin and primary cultures and are likely to represent the different compartments of the vaginal tract.

The cell lines were maintained in keratinocyte serum-free medium (Gifco BRL 17005-042) supplemented with human recombinant EGF (0.1 ng/mL), bovine pituitary extract (0.05 mg/mL), and calcium chloride (0.4 mM) at 37°C with a 5% CO_2_ in air atmosphere.

Adhesion of the Lcr35 was assayed by seeding cell lines in 24-well tissue culture plates at 2.5 × 10^5^ epithelial cells/well and allowing them to grow to complete confluence (10^5^ cells/well). After gentle washing of the cell monolayer, the adhesion capacity of Lcr35 was determined by adding 10^5^ multiplicity of infection (MOI,1), 10^6^ (MOI, 10), and 10^7^ (MOI, 100) bacteria from an overnight culture in de Man, Rogosa, Sharpe (MRS) agar medium. Bacterial cells were previously washed in phosphate buffered saline and resuspended in the cell culture medium. Adhesion was monitored after 1 and 3 hours of incubation carried out at 37°C under 5% CO_2_. The monolayers were washed three times with 1 mL of Dulbecco's phosphate buffered saline, detached by addition of 0.1% TritonX-100 solution and the number of viable bacteria determined by plating serial dilutions of the suspensions onto MRS agar plates. For qualitative analysis, the cell monolayers and the bacteria were methanol fixed and stained by addition of a 10% Giemsa solution.

### 2.2. Growth inhibition of vaginosis-associated pathogens

The effect of Lcr35 on the growth of three pathogens was investigated using the following strains: Candida albicans ATCC10231, *Prevotella bivia* ATCC29303, and *Gardnerella vaginalis* ATCC14018. Coculture assays were performed in either Sabouraud broth (Candida) or brain heart infusion supplemented with yeast extract (1%), maltose (0.1%), glucose (0.1%), and horse serum (10%) (*Prevotella* and *Gardnerella*). Each pathogen (10^8^ UFC) was incubated alone (control) and with the Lcr35 (10^8^ UFC) at 37°C under anaerobic conditions for the two vaginosis-associated bacteria (AnaeroGen, Oxoïd). Aliquots were removed after 4, 8, and 24 hours of incubation, serially diluted and plated on appropriate media (Sabouraud, Gardnerella, or MRS) to determine the bacterial colony counts of both the pathogens and Lcr35. Statistical analyses of the data were performed using the Mann-Whitney test.

## 3. RESULTS

### 3.1. Adhesion of Lcr35 to cervical and vaginal cells

**Figure 1:**
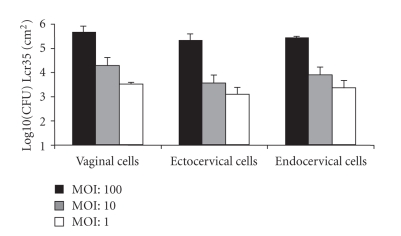
Adherence of *L. rhamnosus* Lcr35 to vaginal, ecto- and endocervical cells. Epithelial cells were incubated with three different bacterial inocula and incubated for 1 hour. The number of viable Lcr35 adhering to the cell monolayer surface was determined by plating onto appropriate media. The data are averages of three independent experiments performed in triplicate. Error bars indicate standard deviations.

**Figure 2:**
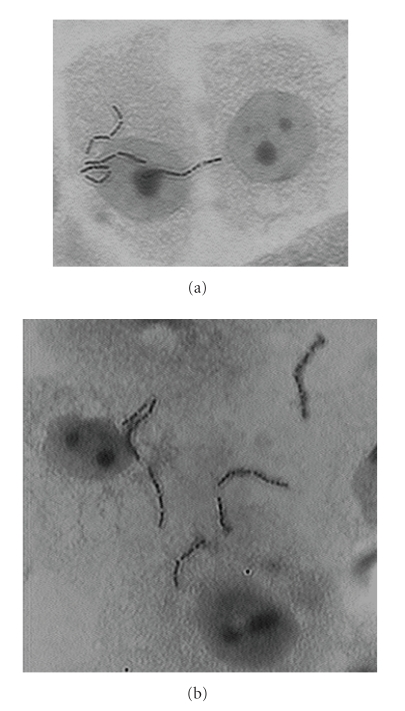
Giemsa-stains from adherence assays performed for the experiment shown in Figure 1 with (a) vaginal and (b) ectocervical cells.

The ability of Lcr35 to adhere to vaginal and cervical cells is shown in Figure 1. Whatever the MOI and the cell line, the probiotic strain was able to adhere to the cell surface monolayer. The highest number of adherent bacteria was observed with the vaginal cell line, with an average of 4.75 10e5 CFU per cm^2^ after 1 hour of incubation. No major difference was observed between the levels of adhesion obtained after 1 hour and 3 hours of incubation (data not shown), suggesting that adhesion occurs rapidly after the initial contact between the cells and the bacteria. Microscopical observations of Giemsa-stained preparation showed typical chains of Lcr35 randomly dispersed on the cell surface (Figure 2).

### 3.2. Growth inhibition of vaginosis-associated pathogens

**Figure 3:**
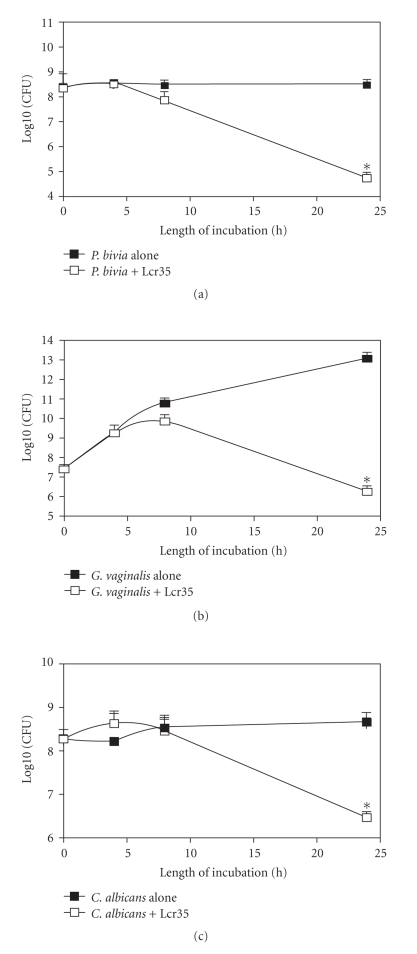
Effect of Lcr35 on the viability of *Prevotella bivia* (a), *Gardnerella vaginalis* (b), and *Candida albicans* (c) as a function of the time of coculture. The pathogen was incubated without (filled square) or with (empty squares) Lcr35 at 37°C for 24 hours and the colony forming unit mL^−1^ was determined after 4, 8, and 24 hours of incubation by plating onto appropriate media. Each value shown is the mean ± SD from three experiments. * : statistically significant differences (*P* = .050, Mann-Whitney test).

The antagonist effect of Lcr35 against three main pathogens, *P. bivia*, *G. vaginalis*, and *C. albicans*, was assessed in coculture assays and compared with the growth ability of each pathogen in the same culture medium (Figure 3). A decrease in the cell division rate of the three microorganisms tested was observed from 4 hours of coincubation. When the viable bacteria in the mixed suspension were counted over a longer period of time, bactericidal activity was detected between 8 and 24 hours of incubation for all pathogens with the *Prevotella* strain being the most susceptible (4-log10 units decrease in the number of viable cells). In no case there was a bactericidal effect against *Lactobacilli*; the number of viable Lcr35 cells was either constant over the incubation period (coculture with *C. albicans*) or increased (coculture with *G. vaginalis* and *P. bivia*) (data not shown).

## 4. DISCUSSION

*Lactobacillus* species in the female urogenital system act as a barrier to infection and contribute to the control of the vaginal microbiota by competing with other microorganisms for adherence to epithelial cells, displacing pathogen biofilm [12, 13], and/or inhibiting the growth of potential pathogens [14–16]. Hence the use of probiotic strains of *Lactobacilli* is potentially interesting both as preventive and curative agents.

Unlike the use of vaginal epithelial cells collected from healthy premenopausal women, assays performed with immortalized epithelial cell lines, which closely resemble the epithelial differentiation patterns of normal human tissues, are more accurate for standardizing tested bacterial adherence and allow comparison of different research approaches. The three epithelial cell lines tested in this study were developed from normal human vagina, ectocervix, and endocervix tissue, and their characteristics closely resembled those of their tissues of origin and primary cultures [11]. We can thus speculate that adhesion assays performed with this material reproduce more faithfully the in vivo situation than experiments performed with any cell line derived from human carcinoma of the lower genital tract mucosa. This is particularly important when comparing bacterial strains belonging to the complex *Lactobacillus* genus that includes bacterial strains with highly specific characteristics. Using these cell lines, we observed specific adhesion of an *L. rhamnosus* strain, Lcr35, previously selected for its probiotic features [8–10]. Adhesion occurred even at a low MOI (1:1) and within less than 1 hour of contact, which corresponds to a highly dynamic process.

Adhesion of Lcr35 to vaginal epithelial cells would allow colonization of the vaginal mucosa and therefore could limit the overgrowth of pathogens, but the second main property of a potential probiotic used as a therapeutic agent against pathogenic microorganisms is direct impairment of their growth. In this study, we demonstrated that Lcr35 showed bactericidal activity against both *P. bivia* and *G. vaginalis* in the range of killing stipulated for the bactericidal activity of antimicrobial activity (>2 log-unit). In a previous study, Atassi et al. demonstrated that the bactericidal activity of *Lactobacilli* toward these two vaginal bacterial pathogens was strain dependent and occurred within the first hours of coculture [14]. In our experiments, a longer incubation time was required to observe bactericidal activity, probably because of the different experimental parameters used in the two assays. We previously showed that the Lcr35 probiotic strain was also able to kill several pathogens [10]. The mechanism(s) underlying this activity has not been elucidated but is likely to be multifaceted and probably includes the production of hydrogen peroxide, lactic acid, and antibacterial compounds. It has been recently shown that *G. vaginalis* organized in biofilms is more resistant to H_2_O_2_ and lactic acid than planktonic cultures [17]. *G. vaginalis* is the predominant species observed within biofilms present on the vaginal epithelium in bacterial vaginosis [18], and Saunders et al. recently showed that strains of *Lactobacilli* were able to disrupt *G. vaginalis* preformed biofilms [13]. It would therefore be interesting to test the biofilm activity of Lcr35 against *G. vaginalis* and to determine if the sessile form of Lcr35 also exhibits antibacterial activity against *G. vaginalis* in mixed biofilm assays.

The antagonist activity of Lcr35 was not limited to bacterial pathogens since the strain was also able to reduce the viability of *C. albicans*. Several strains of *Lactobacilli* have shown inhibitory effects against *C. albicans* [16], which is the species most often associated with candidiasis. By interfering with *Candida* overgrowth in the patients' intestinal or vaginal tract, *Lactobacilli* could provide colonization resistance and maintain low numbers of yeasts, especially when administered together with antibiotics.

Relevant clinical trials have suggested that intravaginal or oral administration of *Lactobacillus* strains is able to increase the numbers of vaginal *Lactobacilli* and restore the vaginal microbiota to normal [7]. Lcr35 was isolated from a human intestinal and not vaginal microbiota and does not belong to the four main species of *Lactobacilli* considered to be predominantly linked to the vaginal microflora, *L. crispatus*, *L. jensenii*, *L. gasseri*, and *L. iners* [4, 19, 20], but it has been shown to survive within the human gastrointestinal tract [9]. Furthermore, Petricevic and Witt recently showed in a clinical study that topical administration of Lcr35 enhances the restoration of the vaginal flora after antibiotic treatment of BV [21]. Thus, it might be an excellent candidate for use as a prophylactic agent, taken orally or applied topically. In vivo studies to evaluate its feasibility as such are in progress.

## 5. CONCLUSION

Maintenance or reconstruction of the normal composition of the vaginal microflora by applying properly selected *Lactobacilli* may be of prophylactic value in preventing or curing genitourinary system infections in women. In the light of our experiments, it seems that the probiotic strain *L. rhamnosus* Lcr35 would be a good candidate as a protective agent against both bacterial vaginosis and *Candida* vaginitis since it was able to adhere to vaginal and cervical cells and to antagonist the growth of vaginosis-associated pathogens. Clinical studies are now required to assess the in vivo efficacy of such a therapy.
